# Balancing innovation and integrity in peer review

**DOI:** 10.1016/j.patter.2024.101061

**Published:** 2024-09-13

**Authors:** Andrew L. Hufton

**Affiliations:** Editor-in-Chief, *Patterns*

## Main text

Peer Review Week is later this month, and this year’s theme is the intersection of innovation and technology. Modern research is digital; so must be peer review. Today, manuscripts are transmitted instantly through secure online reviewing portals rather than being sent through the mail. Peer review is enriched by links to repositories or websites where reviewers can explore data and code in detail. New technologies, however, also create new risks. It is crucial that authors, editors, and reviewers be mindful of these risks and think critically about new technologies to protect the integrity of the peer-review process.

Generative AI tools, like ChatGPT, offer a cautionary example. While these tools are now widely employed by researchers, *Patterns* and all other Elsevier journals strictly forbid their use when reviewing a paper (full policy). The US National Institutes of Health similarly prohibits use during review of grant applications (Lauer et al. 2023, *Review Matters*). We have, however, recently encountered cases where reviewers admitted to using AI tools during review.

It is obvious that creating a “fake” peer review report with an AI tool would be unethical, but not all researchers may understand why it is wrong to use AI merely to refine their own review. Indeed, we know that many researchers now routinely use generative AI to help polish their writing. The answer is simple: confidentiality. As a reviewer, you are being entrusted with private, unpublished content. Sharing that content with a third party, including an external AI tool provider, is a breach of that trust. Generative AI services also learn from content inputted by users, meaning that the details of someone else’s confidential research could, in principle, end up in the AI model in some fashion.

Keep in mind that you are being invited to review a paper because of your scientific expertise, not your beautiful prose. It is okay to submit your report in your own natural writing style, grammar errors and all.

Reviewer anonymity is another area where technologies can either help or create new risks. Online peer-review systems, like the one used by *Patterns*, are designed to be secure and to rigorously protect personal information. At the same time, modern papers often contain links to external websites and resources whose security cannot be guaranteed by the journal. As those pop-ups are consistently reminding us—browsing the web is not inherently anonymous. Web servers can record your IP address and can infer other personal information using tracking technologies like cookies.

Does this mean that we need to rigorously scrub all links from papers? Or admit that anonymity is impossible? No, even if a bad actor did use technology to reveal the identity of a reviewer, ethical norms would limit their ability to benefit from this information. Indeed, in my fourteen years working as a scientific editor, I have never personally encountered a case where a reviewer’s identity was maliciously revealed.

Authors can, nonetheless, take a number of steps to help make their papers more suitable for anonymous peer review. This shows respect for reviewers and increases the chance that they will engage with data and code records outside the main manuscript, which, in my experience, tends to make peer review more constructive. When preparing your paper:

• Share data, code, and models through formal, scholarly repositories, not through a lab website or a personal cloud service.
• If you want to keep files private during peer review, select a repository that offers support for private sharing. Zenodo, OSF, and figshare are popular options with our authors.
• Check that links in your paper are free of tracking codes and that websites to which you link do not request any personal information.

As a reviewer, you can also take some simple steps to help protect your anonymity:

• Switch your browser into private or incognito mode when accessing links in a paper you are reviewing. This provides a reasonable level of protection against inadvertently sharing your identity via cookies or by automatic login to a service you use regularly.
• If you have reason to be particularly concerned about your anonymity, consider using a virtual private network (VPN) or the Tor Browser to mask your IP address.
• If you encounter a login screen while following a link in a paper, contact the journal and ask for help. This is unlikely to be a sign of ill intent, but it is still good to alert the journal in these cases. The journal may also be able to help you access any content you require for your review.

Ultimately, it is the behavior of authors, reviewers, and editors that ensures that peer review is run in an ethical manner. By being mindful of the ethical foundations of scientific peer review, we can ensure that new technologies are used selectively and wisely, enhancing rather than undermining the peer-review process.

